# Clinical and Polysomnographic Features Associated with Poor Sleep Quality in Patients with Obstructive Sleep Apnea

**DOI:** 10.3390/medicina58070907

**Published:** 2022-07-08

**Authors:** Aleksander Kania, Kamil Polok, Natalia Celejewska-Wójcik, Paweł Nastałek, Andrzej Opaliński, Barbara Mrzygłód, Krzysztof Regulski, Mirosław Głowacki, Krzysztof Sładek, Grażyna Bochenek

**Affiliations:** 1 2nd Department of Internal Medicine, Faculty of Medicine, Jagiellonian University Medical College, Jakubowskiego 2, 30-688 Krakow, Poland; kamil.polok@uj.edu.pl (K.P.); natalia.celejewska@uj.edu.pl (N.C.-W.); pnastalek@gmail.com (P.N.); krzysztof.sladek@uj.edu.pl (K.S.); grazyna.bochenek@uj.edu.pl (G.B.); 2 Centre for Intensive Care and Perioperative Medicine, Jagiellonian University Medical College, Wroclawska 1-3, 30-901 Krakow, Poland; 3 Pulmonary Diseases Department, Proszowice District Hospital, Kopernika 13, 32-100 Proszowice, Poland; 4 Department of Applied Computer Science and Modelling, AGH University of Science and Technology, Al. Mickiewicza 30, 30-059 Krakow, Poland; andrzej.opalinski@agh.edu.pl (A.O.); mrzyglod@agh.edu.pl (B.M.); regulski@agh.edu.pl (K.R.); glowacki@metal.agh.edu.pl (M.G.)

**Keywords:** sleep apnea, obstructive sleep apnea, sleep quality, PSQI, polysomnography, heart failure, nocturnal hypoxia, comorbidities, deep sleep

## Abstract

*Background and Objectives*: Poor sleep quality in patients with obstructive sleep apnea (OSA) may be associated with different clinical and polysomnographic features. The aim of this study was to identify features associated with poor sleep quality in OSA patients. *Materials and Methods:* This was a cross-sectional study enrolling patients with OSA confirmed by polysomnography (PSG). In addition to gathering clinical data, patients were assessed using the Pittsburgh Sleep Quality Index (PSQI), the Epworth Sleepiness Scale (ESS), and the Clinical Global Impression Scale. Univariate and multivariable analyses were performed to identify factors associated with an increased risk of poor sleep quality in this population. *Results*: Among 505 enrolled patients (mean age of 57.1 years, 69.7% male) poor quality of sleep (PSQI score ≥ 5) was confirmed in 68.9% of them. Multivariable analysis revealed the following factors associated with poor sleep quality: chronic heart failure (OR 3.111; 95% CI, 1.083–8.941, *p* = 0.035), male sex (OR 0.396; 95% CI, 0.199–0.787, *p* = 0.008), total ESS score (OR 1.193; 95% CI, 1.124–1.266, *p* < 0.001), minimal saturation during sleep (OR 1.034; 95% CI, 1.002–1.066, *p* = 0.036), and N3 percentage of total sleep time (OR 1.110; 95% CI, 1.027–1.200, *p* = 0.009). *Conclusions*: Our study suggests that both the female sex and coexistence of heart failure are independent risk factors for poor sleep quality. Moreover, we hypothesize that nocturnal hypoxia may lead to a misperception of sleep quality and may explain the counterintuitive association between a higher proportion of deep sleep and poor sleep quality.

## 1. Introduction

An adequate quality and amount of sleep are crucial for maintaining proper health and everyday functioning [1]. Poor sleep quality and insufficient duration of sleep may have serious consequences for health, as well as reducing the quality of life [2,3]. Moreover, this may be associated with numerous physical and mental conditions, as well as with sleep disorders. Sleep disorders affect approximately 25% of the general population; thus, poor sleep quality is a common problem [4].

Assessments of sleep quality encompass features such as sleep onset latency, sleep duration, continuity, timing, alertness, and satisfaction [5]. The Pittsburgh Sleep Quality Index (PSQI) is a well-recognized tool for assessing sleep quality [6]. It is based on the patient’s subjective assessment of sleep quality in the preceding month and classifies patients as good or poor sleepers [6].

Obstructive sleep apnea (OSA) is one of the most common sleep disorders [7]. It is associated with the fragmentation of sleep due to repeated arousal linked to apneas. Both extensive sleepiness and poor sleep quality are a consequence of apnea-related sleep fragmentation [8].

Despite common reductions in sleep quality in OSA patients, its formal assessment has rarely been undertaken in this population. The typical approach in daily clinical practice is to assess excessive sleepiness during the day (hypersomnia) using the Epworth Sleepiness Scale (ESS) [9]. There are poor data on risk factors for poor sleep quality in patients with OSA. Therefore, in this study, we aimed to identify clinical and polysomnographic (PSG) features that could be associated with poor sleep quality, assessed using the PSQI in patients with OSA. In order to provide valuable and clinically useful data we comprehensively evaluated a wide array of clinical features, including physical examination features, clinical scales scores, comorbidities, laboratory tests, arterial blood gas analysis, and polysomnographic variables. We are not aware of any previous study that has investigated this issue through such an extensive approach.

## 2. Materials and Methods

### 2.1. Patients

This was a cross-sectional study including 505 consecutive patients admitted to the sleep disorders laboratory of the Department of Pulmonology at Jagiellonian University Medical College, Krakow, Poland, in whom OSA was confirmed by PSG. Only patients with a diagnosis of OSA based on American Academy of Sleep Medicine criteria, i.e., apnea–hypopnea index (AHI) > 5 [10], and who provided written informed consent to participate in the study were included. The study was conducted in accordance with the Declaration of Helsinki, and the study protocol was approved by the Ethics Committee of Jagiellonian University.

### 2.2. Data Collection

On admission to the hospital, a detailed interview and physical examination were performed by a member of the study personnel. Detailed data regarding demographic characteristics, clinical features (comorbidities, medications, symptoms), body measurements (height, weight, circumferences of neck, hips, and waist), as well as vital signs (blood pressure, heart rate, and saturation) were gathered. Moreover, each patient was assessed using the following scales: the Pittsburgh Sleep Quality Index, Clinical Global Impression Scale, and Epworth Sleepiness Scale. All clinical indexes utilized in this study are described in detail below. Moreover, arterial blood gas analysis was performed in each study participant. All of the gathered data were stored in a cloud-based, password-protected, and encrypted database created and maintained by a team of computer scientists from AGH University of Science and Technology in Krakow.

### 2.3. Scales

#### 2.3.1. The Pittsburgh Sleep Quality Index

Sleep quality over the previous month was evaluated using the PSQI [6]. The questionnaire includes seven components, each one pertaining to a major aspect of sleep: subjective sleep quality, sleep latency, sleep duration, sleep efficiency, sleep disturbances, use of sleep medication, and daytime dysfunction. The 7 component scores are added to obtain a global score ranging from 0 to 21, with higher scores indicating worse sleep quality. A total score > 5 indicates poor quality of sleep [6,11]. Based on this cutoff score, participants were divided into 2 groups: those with good sleep quality (PSQI ≤ 5) and those with poor sleep quality (PSQI > 5). Permission to use the PSQI questionnaire for the purpose of this study was obtained from the developer.

#### 2.3.2. The Clinical Global Impression Scale

Assessment of the clinician’s view of the patient’s global functioning was performed using the Clinical Global Impression (CGI) scale [12]. This scale provides an overall clinician-determined summary measure that takes into account all available information, including knowledge of the patient’s history, psychosocial circumstances, symptoms, behavior, and the impact of the symptoms on the patient’s ability to function. The CGI Severity scale (CGI-S) asks the clinician one question: “Considering your total clinical experience with this particular population, how ill is the patient at this time?”; this is rated on a 7-point scale: 1 = normal, not at all ill; 2 = borderline mentally ill; 3 = mildly ill; 4 = moderately ill; 5 = markedly ill; 6 = severely ill; and 7 = among the most extremely ill patients.

#### 2.3.3. The Epworth Sleepiness Scale

The presence of hypersomnia was assessed with the ESS [9]. The ESS consists of 8 self-rated items (each scored from 0 to 3 points) that measure a subject’s habitual likelihood of dozing or falling asleep in common situations of daily living: sitting and reading, watching TV, sitting, being inactive, in a public place (e.g., in a meeting, theater, or dinner event), as a passenger in a car for 1 hour or more without stopping for a break, lying down to rest when circumstances permit, sitting and talking to someone, sitting quietly after a meal without alcohol, and in a car while stopped for a few minutes in traffic or at a light. The total ESS score represents the sum of individual items, and ranges from 0 to 24. The higher the ESS score, the higher a person’s propensity to sleep in everyday life. An ESS score > 10 represents excessive daytime sleepiness.

### 2.4. Polysomnography

All patients underwent full-night diagnostic PSG in a hospital setting. Recorded signals included electroencephalography derived from F3, F4, C3, C4, O1, and O2 electrodes, electro-oculogram, electrocardiogram, and mentalis electromyography recorded with surface electrodes, airflow measured with a thermistor and nasal pressure transducer, and snoring recorded with the microphone attached to the neck skin. Respiratory movements were monitored with inductance bands placed around the thorax and abdomen. Blood oxygen saturation (SpO_2_) was monitored with finger oximetry. During the night, patients were videotaped with an infrared camera, recorded on an Alice 6 Diagnostic Sleep System (Philips North America Corporation, Andover, MA, USA). Respiratory events were scored according to the guidelines of the American Academy of Sleep Medicine [13]. Polysomnography outcomes included total sleep time, sleep efficiency, sleep architecture, AHI, mean saturation, minimal saturation, total sleep time spent with SpO_2_ < 90% (T90), oxygen desaturation index (ODI), and body position during sleep.

### 2.5. Other Physical Measurements

Arterial blood gas samples were collected at 6 a.m. from the radial artery in heparinized syringes and gauged for 30 min with an auto-analyzer (ABL90 FLEX PLUS, Radiometer, Copenhagen, Denmark) using an electrode method, also considering lactate levels. Blood samples were obtained while patients were awake, fasting, and in a supine position. A value > 0.8 mmol/L was considered as an elevated lactate level.

### 2.6. Statistical Analysis

Quantitative variables are reported as the mean (SD), whereas qualitative variables, are reported as the number and percentage of patients. Univariate associations between the potential predictors of poor sleep quality and the actual occurrence of poor quality of sleep (defined as PSQI > 5) were assessed with odds ratios (ORs); 95% confidence intervals (95%CIs) for ORs are also reported. Multivariable analysis of potential predictors of poor sleep quality was conducted using logistic regression. The model included the following independent variables selected according to the literature and the authors’ best knowledge: sex, age, body mass index (BMI), EES score, C-GIS, arterial hypertension, ischemic heart disease, atrial fibrillation, other arrhythmias, stroke, atherosclerosis of the lower limbs, type 2 diabetes, sleep effectiveness, AHI, ODI, minimal saturation, T90, sleep stages, arterial blood gas results, arterial lactate level, oxygen saturation measured with pulse oximetry (SpO_2_), systolic blood pressure, diastolic blood pressure, and pulse. Qualitative variables were incorporated in the logistic regression using the usual dummy variable coding scheme. Only observations (patients) with no missing data were included in the multivariable analysis. The significance level for all statistical tests was set at 0.05. R 4.1.2. was used for computations [14].

## 3. Results

The demographic, clinical, and PSG data of the studied group are presented in Table 1.

Of the 505 enrolled participants, 69.7% were male. The mean age of the study group was 57.1 years (SD = 12.6). The mean body mass index was 33.3 kg/m^2^ (SD = 8.4). The mean total ESS score was 8.8 (SD = 4.8). The global assessment of disease severity based on the CGI-S scale revealed that only 9.5% of patients were classified into the normal group. The most common comorbidities were hypertension, followed by type 2 diabetes, ischemic heart disease, chronic heart failure, atrial fibrillation and other arrhythmias, stroke, and atherosclerosis of the lower limb. Out of the PSG parameters, the mean sleep effectiveness was 81.4% (SD = 11.7); the mean AHI was 42.7 (SD = 24.3); and the mean ODI was 44.8 (SD = 33.9) per hour of sleep. PSG revealed mild, moderate, and severe OSA in 76 (15.0%), 109 (21.6%), and 320 (63.4%) patients, respectively.

Patients with a poor quality of sleep constituted 68.9% of study group (Table 2). In the group of poor sleepers, there were significantly fewer males (63.8% vs. 82.8%, *p* < 0.001) and patients had a significantly higher body mass index (33.9 vs. 32.7, *p* < 0.001) and total ESS score (9.6 vs. 6.9, *p* < 0.001), as compared with good sleepers (Table 2).

There were significant differences between groups in terms of the clinician’s assessment of the illness according to the CGI-S scale. Among comorbidities, hypertension and chronic heart failure were significantly more frequent in patients with poor sleep quality than in those with good sleep quality, but the difference for chronic heart failure showed only borderline significance (11.8% vs. 5.7%, *p* = 0.052). Among PSG parameters, only the percentage of N3 sleep was significantly higher in poor versus good sleepers (9.3% vs. 7.3%, *p* = 0.015) (Table 3).

Univariate regression analysis revealed that the male sex was related with decreased odds of being in the group of poor sleepers by 63% (Table 2). Every 1-point increase in the ESS total score was associated with increased odds of being in the poor sleep quality group by 15% (OR 1.145; 95% CI 1.094–1.198, *p* < 0.001). The clinician’s assessment of illness according to the CGI-S scale as “moderately ill” and “markedly ill” was associated with an increased chance of being in the poor sleep quality group by 2.5-fold (OR 2.479; 95% CI 1.237–4.967, *p* = 0.042) and 2.7-fold (OR 2.722; 95% CI 1.228-6.035, *p* = 0.042), respectively. Arterial hypertension and chronic heart failure were associated with increased odds of poor quality of sleep by 63% (OR 1.631; 95% CI 1.107–2.404, *p* = 0.017) and by 2.1-fold (OR 2.196; 95% CI 1.040–4.638, *p* = 0.052), respectively. Among PSG parameters (Table 3), every 1% increase in N3 stage of sleep was related with an increased risk of being in the poor sleep quality group by 3% (OR 1.033; 95% CI 1.008–1.058, *p* = 0.015).

The multivariable regression analysis revealed that the male sex was associated with a 60% lower risk of poor sleep quality (Table 4). An increase in the total ESS score by 1 point was associated with increased odds of poor sleep quality by 19% (OR 1.193; 95% CI 1.124–1.266, *p* < 0.001). Chronic heart failure was associated with a 3.1-fold higher risk of poor sleep quality (OR 3.111; 95% CI 1.083–8.941, *p* = 0.035). Among the PSG parameters, every 1% increase in minimal saturation and every 1% increase in the percentage of N3 sleep was associated with an increased risk of poor sleep quality by 3% (OR 1.034; 95% CI 1.002–1.066, *p* = 0.036) and 11% (OR 1.110; CI 1.027–1.200, *p* = 0.009), respectively.

## 4. Discussion

Our cross-sectional study including over 500 patients with PSG-confirmed OSA revealed that the risk of poor sleep quality was significantly associated with clinical parameters such as chronic heart failure, sex, and hypersomnia, as well as PSG parameters such as N3 sleep and minimal saturation. To the best of our knowledge, we are the first to comprehensively analyze clinical and polysomnographic parameters that affect sleep quality among OSA patients. We found that heart failure was the only comorbidity assessed in our study that was linked with poor sleep quality. The diagnosis of chronic heart failure was associated with a threefold higher risk of poor sleep quality. Heart failure is more common in patients with OSA than in healthy individuals; patients with OSA are more likely to suffer from heart failure [15,16]. OSA can potentially influence changes in heart structure [17]. In our study, almost 10% of patients with OSA had a history of chronic heart failure, and heart failure was twice as frequent among patients with poor sleep quality. It was reported that sleep-related breathing disorders occur in 40% to 50% of patients with symptomatic heart failure [18]. Sleep-related breathing disorders in heart failure include primarily central sleep apnea, although OSA is also common [16]. Awotidebe et al. [19] reported a significantly worse sleep quality in patients with heart failure compared with healthy controls. In a Spanish study by Jorge-Samitier et al. [20] on a group of 203 patients admitted for decompensated heart failure, poor sleep quality was noted in 73.3%. Javadi et al. [21] revealed poor sleep quality in 91% of hospitalized patients with heart failure. Worsening sleep quality in heart failure may be associated with various conditions, such as nocturia, awakening with inability to fall asleep again, or restless leg syndrome [21,22]. In the Rotterdam study, the OR for poor sleep quality in patients with heart failure was 2.63 [23]. The OR of 3.11 in our study indicates that the coexistence of heart failure with OSA was associated with an increased risk of poor sleep quality in these patients. Heart failure may worsen and alter sleep-related breathing disorders, which may lead to deteriorations in sleep quality. Therefore, an OSA patient reporting poor sleep quality should prompt investigation to exclude heart failure, and if heart failure has already been diagnosed, efforts should be made to optimize its management.

Sex was another factor associated with sleep quality in our study. Male sex was associated with reduced risk of poor sleep quality. Thus, the risk of poor sleep quality was higher in women than in men. The effect of sex on sleep quality was reported in previous population studies. In an Australian study of young adults, Fatima et al. [24] showed that females had almost twofold higher odds of poor sleep quality than males. The risk remained significant even after adjustment for the role of sociodemographic factors, lifestyle, and medical problems. In the study by Madrid-Valero et al. [25], assessing patients aged 43 to 71 years, women were almost twice as likely as men to have poor quality of sleep. Similar findings were reported in a population-based cross-sectional Chinese study including almost 27,000 participants [26]. Interestingly, data from the Korean survey showed contrasting results: participants in the poor sleep quality group were more likely to be male [2]. Few studies have specifically assessed the effect of sex on sleep quality in patients with OSA. Kim et al. [27] reported no such link, but their population was limited to patients with obesity. Our study, on the other hand, included both patients with obesity and those of normal weight. Miyahara et al. [28] described poorer sleep quality in women; however, they assessed the risk of OSA only on the basis of the Berlin questionnaire and did not confirm the diagnosis by PSG. It is known that women and men differ in terms of the clinical symptoms of OSA. Valipiur et al. [29] revealed that women with OSA complained significantly more often of insomnia, restless legs, depression, nightmares, palpitations at night, and hallucinations than men, which may affect the quality of sleep. Moreover, women with OSA more often reported morning fatigue and headache as well as they more often used sleeping medications than men [30]. Their subjective assessment of sleep quality may be significantly affected by these factors. Importantly, the sex of patients with OSA could potentially determine how complete the PSQI questionnaire was. Such an assumption can be made through an analogy to the results of the Sleep Heart Health Study, showing that, even though women experienced sleepiness as often as men, they were less likely to have an ESS score > 10 [31]. If similar relationships pertain to sleep quality assessments and PSQI scores, the increased risks of poor sleep quality among women with OSA observed in our study may even be underestimated. Finally, our observations corroborate the results of the study by Awotidebe et al. [19], who reported a higher risk of poor sleep quality among women with heart failure as compared with men.

Even though a strong causal association between poor sleep quality and sleepiness seems to be logical, the available literature is inconsistent regarding this issue. Our study, based on a relatively large and homogenous sample of OSA patients, suggests that such relationship exists and may be of practical value, i.e., each one-point increase in the ESS score was associated with increased risk of poor sleep quality. Although hypersomnia is the most typical symptom reported by patients with OSA, its severity does not correlate with the severity of OSA itself. Among patients with severe OSA, hypersomnia was reported only in 35% [32]. In a study by Buysee et al. [11], assessing relationships between the results of PSQI, ESS, and clinical/PSG measures in a community sample, there was a weak correlation between the ESS and PSQI scores. Gelaye et al. [33] assessed students from four culturally different countries and showed considerable differences in the strength of correlation between the ESS and PSQI scores, suggesting limited usefulness of the ESS scores to evaluate sleep quality. In our study, the PSQI score was significantly related to the ESS score, confirming that patients with OSA constitute a specific population with a particularly strong association between sleepiness and quality of sleep. Studies in other populations, e.g., hemodialysis patients, showed that the PSQI score is not always strongly correlated with the ESS score, because these patients did not show hypersomnia despite poor sleep quality [34]. It is possible that in patients with OSA the same or closely linked factors are responsible for hypersomnia and reductions in sleep quality.

The study by Buysee et al. [11] revealed that symptoms assessed by the ESS and PSQI are not correlated with objective sleep features measured by PSG. Basunia et al. [35] reported that among patients with OSA, symptoms such as excessive fatigue or sleepiness, falling asleep during the day, waking up tired, trouble paying attention, snoring, and insomnia were significantly related to a decreased percentage of N3 sleep. Conversely, in our study, increasing N3 percentages of total sleep time were linked to higher risks of poor sleep quality. However, this finding requires further research. Veauthier et al. [36] examined a group of patients with untreated sleep-related breathing disorders, revealing that the percentage of deep sleep measured by PSG was higher in good sleepers compared with poor sleepers. In a previous study on the same population, Veauthier [37] reported that the AHI in their study group was 32.5/h. Although our study group was similar in terms of age and sex, the mean AHI in our study was higher and reached 42.7/h. This discrepancy may be because we included patients with a more severe form of OSA. The simple truth that a greater amount of deep sleep translates into higher subjective quality does not seem to apply to patients with OSA. This conclusion is similar to another paradox described by Wu et al. [38], who reported that more severe hypoxemia was associated with better subjective sleep quality in patients with OSA. Similarly, in our study, even a slight increase in minimum saturation was associated with an increased risk of poor sleep quality. Based on this, it may be concluded that the sleep quality in patients with OSA depends on multiple factors affecting subjective assessments, including hypoxia. Therefore, the greater the desaturation, the higher the odds that a patient with OSA will develop disorders, leading to misperceptions of sleep quality. Perhaps severe hypoxia, despite a reduced N3 percentage of total sleep time, affects the brain to such an extent that patients are not aware of sleep disturbances, which is reflected in their responses in the PSQI questionnaire. In our study, more than 60% of patients had severe OSA, and 15% had a mild disorder. Therefore, we mainly assessed patients in whom hypoxia appeared to be an important factor impacting the perception of sleep quality, which may have affected our results.

The strengths of our study include the relatively large cohort of OSA patients with a comprehensive assessment of clinical and polysomnographic characteristics. However, the study is not without limitations. First, the generalizability of the presented results is limited by the single-center character of the study and the predominance of severe OSA, which does not reflect the distribution of the disease severity in the general population. Second, we did not assess several parameters potentially affecting sleep quality, such as the use of medications. Third, we used the total PSQI score without consideration of the individual domains. However, this was a conscious decision, because we assumed that the topic of our study was the overall subjective assessment of sleep quality by the patient and not the assessment of individual domains. Additionally, we did not evaluate the physio-pathological mechanisms responsible for the causal relationships between individual clinical and polysomnographic features and sleep quality. Finally, comorbidities were diagnosed on the basis of medical history and available clinical records, and no diagnostic tests were performed.

## 5. Conclusions

This cross-sectional study, which included more than 500 OSA patients, has described factors associated with poor sleep quality using a comprehensive analysis of clinical, laboratory, and polysomnographic data. Both univariable and multivariable analyses demonstrated that the risk of poor sleep quality is over twice as high among women compared with men; however, we speculate that this could be an underestimation. Other factors associated with a higher risk of poor sleep quality were the coexistence of chronic heart failure and increasing daily somnolence, assessed using the ESS. Finally, we observed a seemingly paradoxical association between a decreased quality of sleep and an increasing proportion of deep sleep. From a practical point of view, it seems that women and patients with coexisting heart failure are at particularly high risk of being categorized in the poor sleep quality group and require in-depth assessments of sleep quality in everyday clinical practice. Another clinically relevant take-home message is the potential utility of the ESS scale in the identification of patients at risk of poor sleep quality. Further research on the early identification and optimal management of sleep quality in this population is warranted.

## Figures and Tables

**Table 1:** Demographic, clinical, and polysomnographic characteristics of the study group.

Parameter	Total (*n* = 505) M (SD) or *n* (%)
Male sex	352 (69.7)
Age, years	57.1 (12.6)
BMI, kg/m^2^	33.3 (8.4)
Scales	
Total ESS score	8.8 (4.8)
CGI-S—normal	48 (9.5)
CGI-S—borderline	87 (17.2)
CGI-S—mildly ill	150 (29.7)
CGI-S—moderately ill	134 (26.5)
CGI-S—markedly ill	72 (14.3)
CGI-S—severely ill	14 (2.8)
Comorbidities	
Arterial hypertension	326 (64.6)
Chronic heart failure	50 (9.9)
Ischemic heart disease	73 (14.5)
Atrial fibrillation	45 (8.9)
Other arrhythmias	19 (3.8)
Stroke	17 (3.4)
Atherosclerosis of the lower limbs	13 (2.6)
Type 2 diabetes	123 (24.4)
Medications	
ACE-I/ARB	275 (54.5)
Calcium channel blocker	131 (25.9)
Acetylsalicylic acid	109 (21.6)
Beta-blocker	221 (43.8)
Insulin	30 (5.9)
Oral antidiabetic drug	112 (22.2)
Oral anticoagulants ^a^	54 (10.7)
Polysomnography	
Sleep effectiveness, %	81.4 (11.7)
AHI, events/h	42.7 (24.3)
ODI, events/h	44.8 (33.9)
Minimal saturation, %	77.4 (12.4)
T90, %	47.9 (71.6)
Sleep stage N1, %	41.1 (13.9)
Sleep stage N2, %	34.3 (12.7)
Sleep stage N3, %	14.9 (8.8)
Sleep stage REM, %	9.6 (6.5)
Other tests	
pO_2_, mmHg	76.8 (13.1)
pCO_2_, mmHg	39.4 (4.1)
HCO_3_^−^, mmol/L	25.5 (2.2)
Lactate, mmol/L	1.1 (0.5)
pH	7.42 (0.03)
SpO_2_, %	95.4 (8.8)
SBP, mmHg	137.4 (22.6)
DBP, mmHg	82.1 (14.2)
Pulse, beats/min	78.5 (13.6)

^a^ These include both vitamin K antagonists and non-vitamin K antagonists; M and SD are used to represent the mean and standard deviation, respectively. Abbreviations: ACE-I, angiotensin-converting enzyme inhibitors; AHI, apnea–hypopnea index; ARB, angiotensin receptor blocker; BMI, body mass index; CGI-S, the Clinical Global Impression Severity scale; DBP, diastolic blood pressure; ESS, Epworth Sleepiness Scale; ODI, oxygen desaturation index; REM, rapid eye movement; SBP, systolic blood pressure; SpO_2_, oxygen saturation measured with pulse oximetry; T90, total sleep time spent with SpO_2_ < 90%.

**Table 2:** Differences in the demographic and clinical parameters according to the quality of sleep measured using the PSQI, as well as associations of the parameters with poor sleep quality.

Parameter	PSQI ≤ 5 ^a^ (*n* = 157)	PSQI > 5 ^a^ (*n* = 348)	*p* Value	OR	95% CI
Male sex ^b^ Female sex	130 (82.8) 27 (17.2)	222 (63.8) 126 (36.2)	<0.001	0.366 Ref.	0.229–0.585 Ref.
Age, years	55.6 (13.6)	57.7 (12.1)	0.073	1.014	0.999–1.029
BMI, kg/m^2^	32.7 (7.0)	33.9 (8.2)	<0.001	1.022	0.996–1.048
ESS total score	6.9 (4.3)	9.6 (4.8)	<0.001	1.145	1.094–1.198
CGI-S—normal	21 (13.4)	27 (7.8)	0.042	1.0	
CGI-S—borderline	29 (18.5)	58 (16.7)		1.556	0.754–3.208
CGI-S—mildly ill	54 (34.4)	96 (27.6)		1.383	0.714–2.677
CGI-S—moderately ill	32 (20.4)	102 (29.3)		2.479	1.237–4.967
CGI-S—markedly ill	16 (10.2)	56 (16.1)		2.722	1.228–6.035
CGI-S—severely ill	5 (3.2)	9 (2.6)		1.400	0.408–4.804
Arterial hypertension	89 (56.7)	237 (68.1)	0.017	1.631	1.107–2.404
Chronic heart failure	9 (5.7)	41 (11.8)	0.052	2.196	1.040–4.638
Ischemic heart disease	16 (10.2)	57 (16.4)	0.09	1.726	0.957–3.114
Atrial fibrillation	14 (8.9)	31 (8.9)	1	0.999	0.516–1.935
Other arrhythmias	6 (3.8)	13 (3.7)	1	0.977	0.364–2.618
Stroke	6 (3.8)	11 (3.2)	0.909	0.821	0.298–2.262
Atherosclerosis of the lower limbs	4 (2.6)	9 (2.6)	1	1.015	0.308–3.349
Type 2 diabetes	30 (19.1)	93 (26.7)	0.083	1.544	0.971–2.454

M (SD) or *n* (%); M and SD are used to represent the mean and standard deviation, respectively. ^a^ Poor quality of sleep assessed with the PSQI: global score > 5. ^b^ Note that poor sleep quality was observed in 63.1% of males and 82.4% of females. Abbreviations: see Table 1.

**Table 3:** Results of polysomnography, arterial blood gas test, and physical examination in the patient groups according to the quality of sleep measured using the PSQI and associations of the parameters with poor sleep quality.

Parameter	PSQI ≤ 5 ^a^ (*n* = 157)	PSQI > 5 ^a^ (*n* = 348)	*p* Value	OR	95% CI
Sleep effectiveness, %	81.4 (11.4)	81.4 (11.9)	0.851	1.000	0.984–1.016
AHI, events/h	42.7 (24.5)	42.7 (24.3)	0.97	1.000	0.992–1.008
ODI, events/h	42.3 (28.9)	45.9 (36.0)	0.408	1.003	0.997–1.009
Minimal saturation, %	77.8 (11.6)	77.2 (12.8)	0.408	0.996	0.980–1.011
T90, %	40.3 (60.3)	51.3 (76.0)	0.372	1.002	0.999–1.005
Sleep stage N1, %	42.6 (12.7)	40.4 (14.4)	0.119	0.989	0.975–1.003
Sleep stage N2, %	34.4 (12.3)	34.3 (12.9)	0.74	1.000	0.985–1.015
Sleep stage N3, %	13.4 (7.3)	15.6 (9.3)	0.015	1.033	1.008–1.058
Sleep stage REM, %	9.7 (6.4)	9.6 (6.5)	0.917	0.999	0.969–1.029
pO_2_, mmHg	77.8 (13.0)	76.3 (13.1)	0.219	0.991	0.977–1.006
pCO_2_, mmHg	39.4 (3.6)	39.4 (4.3)	0.904	1.001	0.956–1.048
HCO_3_^−^, mmol/L	25.3 (1.4)	25.6 (2.0)	0.16	1.095	0.984–1.218
Lactate, mmol/L	1.1 (0.5)	1.2 (0.6)	0.04	1.462	0.999–2.141
pH	7.42 (0.0)	7.42 (0.0)	0.093	1.065	0.991–1.145
SpO_2_, %, mean (SD)	94.6 (13.4)	95.7 (5.7)	0.04	1.013	0.993–1.034
SBP, mmHg, mean (SD)	136.4 (17.7)	139.4 (19.5)	0.212	1.009	0.998–1.019
DBP, mmHg, mean (SD)	81.2 (11.2)	83.4 (12.6)	0.088	1.015	0.999–1.032
Pulse, beats/min, mean (SD)	76.3 (15.7)	79.4 (12.4)	0.088	1.017	1.003–1.032

M (SD) or n (%); M and SD are used to represent the mean and standard deviation, respectively. ^a^ Poor quality of sleep assessed with the PSQI: global score > 5. Abbreviations: see Table 1. Conversion factors to SI units are as follows: millimeter of mercury to kilopascal, multiply by 0.133.

**Table 4:** Multivariate logistic regression analysis of significant predictors of poor sleep quality.

Parameter	*p* Value	OR	95% CI
Male sex	0.008	0.396	0.199–0.787
Total ESS score	<0.001	1.193	1.124–1.266
Chronic heart failure	0.035	3.111	1.083–8.941
Minimal saturation, %	0.036	1.034	1.002–1.066
Sleep stage N3, %	0.009	1.110	1.027–1.200

Abbreviations: see Table 1.

## Data Availability

The data that support the findings of this study are available from the corresponding author upon reasonable request. The data are not publicly available due to information that could compromise the privacy of research participants.
